# Filter-Dense Multicolor Microscopy

**DOI:** 10.1371/journal.pone.0119499

**Published:** 2015-03-04

**Authors:** Siavash Kijani, Ulf Yrlid, Maria Heyden, Malin Levin, Jan Borén, Per Fogelstrand

**Affiliations:** 1 Wallenberg Laboratory, Department of Molecular and Clinical Medicine, University of Gothenburg, Gothenburg, Sweden; 2 Department of Microbiology and Immunology, Institute of Biomedicine, The Mucosal Immunobiology and Vaccine Center (MIVAC), University of Gothenburg, Gothenburg, Sweden; German Cancer Research Center, GERMANY

## Abstract

Immunofluorescence microscopy is a unique method to reveal the spatial location of proteins in tissues and cells. By combining antibodies that are labeled with different fluorochromes, the location of several proteins can simultaneously be visualized in one sample. However, because of the risk of bleed-through signals between fluorochromes, standard multicolor microscopy is restricted to a maximum of four fluorescence channels, including one for nuclei staining. This is not always enough to address common scientific questions. In particular, the use of a rapidly increasing number of marker proteins to classify functionally distinct cell populations and diseased tissues emphasizes the need for more complex multistainings. Hence, multicolor microscopy should ideally offer more channels to meet the current needs in biomedical science. Here we present an enhanced multi-fluorescence setup, which we call Filter-Dense Multicolor Microscopy (FDMM). FDMM is based on condensed filter sets that are more specific for each fluorochrome and allow a more economic use of the light spectrum. FDMM allows at least six independent fluorescence channels and can be applied to any standard fluorescence microscope without changing any operative procedures for the user. In the present study, we demonstrate an FDMM setup of six channels that includes the most commonly used fluorochromes for histology. We show that the FDMM setup is specific and robust, and we apply the technique on typical biological questions that require more than four fluorescence microscope channels.

## Introduction

Immunofluorescence microscopy—microscopic detection of proteins using fluorochrome-labeled antibodies—is a powerful approach to analyze the spatial localization of proteins in tissues. The technique can be used for detection of multiple proteins within the same sample (multicolor microscopy) and is therefore a unique method for co-localization analyses and tissue characterization. However, current multicolor microscopy is restricted to a maximum of four fluorochrome channels. Since one channel is often designated for nuclei staining, it follows that only three channels are left for immunodetection. This limits the ability of multicolor microscopy to address common scientific questions that involve multiple cell markers, for example the function of specific cell subsets in tissues (e.g. macrophage subsets [1], T-cell subsets [2], and dendritic cell subsets [3]), or how cells interact in multicellular structures (e.g. in germinal centers [4] and in atherosclerotic lesions [5]). Although many scientific questions can be answered by the use of other methods, such as flow cytometry and qPCR, only microscopy keeps the tissue architecture intact and thereby reveals the tissue context in which cells act. Hence, it would be beneficial if multicolor microscopy could offer more fluorescence channels to keep in line with the rapid progress in biomedical science.

The prevalent system for separation of fluorochrome signals in fluorescence microscopy is based on filter sets [6], which contain an excitation light filter, a beam splitter, and an emission light filter. The excitation filter determines the wavelength interval at which the fluorochrome is excited, and the emission filter determines the wavelength interval at which the emitted signal is collected. By selection of excitation and emission filters, the filter set can be rather specific for a particular fluorochrome. However, the excitation and emission light spectra of fluorochromes are broad, which limits the number of fluorochromes that can be separated from each other by current filter sets. This can be improved by the addition of computer-based signal deconvolution, where bleed-through signals are subtracted using mathematical algorithms [7,8], or color addition, where each epitope is labeled with two or more fluorochromes that together give rise to a new color [9]. However, both these methods are complicated, and they are rarely seen in research and laboratory medicine.

In this study we chose a different and more user-friendly strategy to increase the number of fluorescence channels, which we call Filter-Dense Multicolor Microscopy (FDMM). FDMM is based on condensed filter sets and a selected panel of fluorochromes that together facilitate a more economic use of the light spectra. The FDMM setup presented here has six independent fluorescence channels and includes the most common fluorochromes used in histology.

## Results

### The strategy and design of the FDMM setup

**Fig 1:**
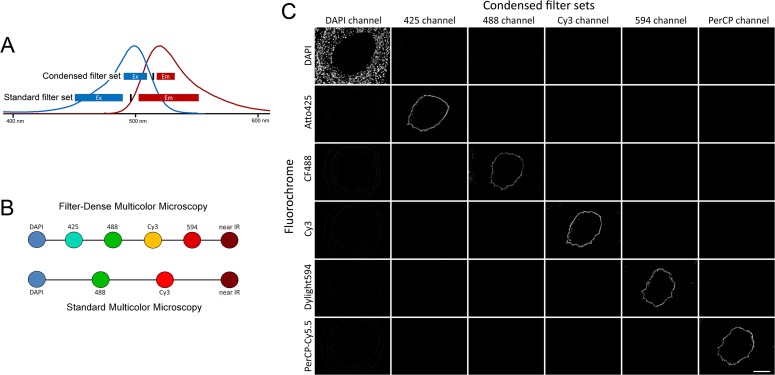
The strategy and specificity of FDMM. *(A)* Example of a condensed filter set compared with a standard filter set (Carl Zeiss, filter set #38) for the 488 channel. The curves show the excitation (blue line) and emission (red line) light spectra of the fluorochrome AF488. Blue rectangles, excitation filter interval; red rectangles, emission filter interval; vertical black line, beam splitter. *(B)* By the use of condensed filter sets, the density of fluorescence channels is increased in the FDMM setup compared with standard multicolor microscopy. *(C)* Each filter set in the FDMM setup is specific for its corresponding fluorochrome. Six tissue sections from a blood vessel were immunolabeled for CD31 (endothelium) with different fluorochromes or nuclei stained with DAPI. Each filter set (columns) received signals from only one fluorochrome (rows). Scale bar, 100 μm. Exposure times: DAPI channel (11 ms), 425 channel (30 ms), 488 channel (63 ms), Cy3 channel (20 ms), 594 channel (54 ms), PerCP channel (17 ms). Objective: x20/0.75.

To increase the number of fluorescence channels, we condensed each filter set so that they were more specific for each fluorochrome and occupied a smaller portion of the total light spectrum (an example is shown in Fig. 1A). The filter sets were carefully chosen so that the excitation filter interval for fluorochrome 1 did not activate the adjacent fluorochrome 2, and the emission filter interval of fluorochrome 2 did not receive emission signal from fluorochrome 1. This strategy allowed separation of fluorochromes with substantial spectral overlap, and hence increased the number of fluorochrome channels within the visible/near infrared light spectrum (Fig. 1B). For illumination, we chose to use a standard mercury lamp and we built the setup around the most commonly used fluorochromes for microscopy, i.e. DAPI, 488 dyes, Cy3 and 594 dyes. The final fluorochrome panel and corresponding filter set arrangements are presented in S1 Fig. and S1 Table. All selected fluorochromes in the FDMM setup are bright and photostable. Each condensed filter set works for many fluorochromes with similar spectra. Examples of fluorochromes that fit into the FDMM setup are presented in Table 1.

**Table 1:** Examples of fluorochromes that fit in the FDMM setup.

Filter set	Examples of suitable fluorochoromes
DAPI	DAPI*, Dylight350, CF350, AF350, AMKA
425	Atto425*, Sytox blue*
488	CF488*, AF488*, Dylight488*, Atto488*, FITC*, eGFP, CFSE*
Cy3	Cy3*, CF543*, AttoRho6G, AF546, Dylight549
594	CF594*, AF594*, Dylight594*, Atto590, Atto594*
PerCP	PerCP-Cy5.5*, PerCP-eFlur710*, PerCP*

* fluorochromes that were tested in the FDMM setup

### The FDMM setup generates specific signals from each fluorochrome and can be run with a standard fluorescence microscope system

The FDMM setup was applied to a standard fluorescence microscope simply by changing the filter cubes. The specificity of each channel in the FDMM setup was verified in tissue sections from blood vessels. We immunolabeled the endothelium (the innermost cell layer) with different fluorochromes and showed that each filter set received signals from only one fluorochrome (Fig. 1C). When exposure times used in Fig. 1C were increased five times for each channel, the specificity remained (S2 Fig.), demonstrating that the FDMM setup is robust. The signal quality from the setup was also verified using a spectra viewer program (Semrock SearchLight). Calculated collected fraction of total emission signal, signal-to-noise ratios, signal-to-bleed-through ratios of the FDMM filter sets are presented in S2 Table. Corresponding data using standard filter sets from Chroma Technology Corp and Carl Zeiss Microscopy are shown in S3 Table and S4 Table.

**Fig 2:**
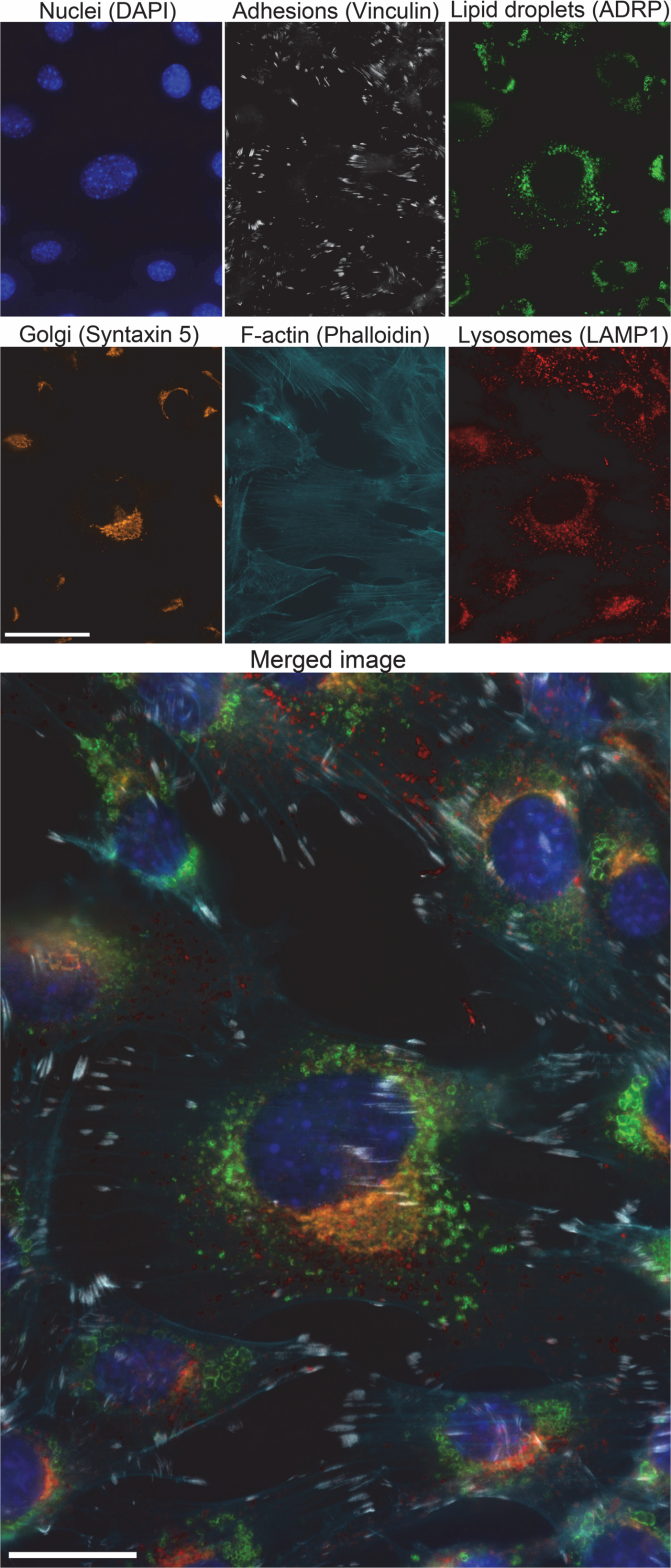
Six channel FDMM for subcellular staining of cultured NIH cells. Cultured NIH cells immunolabeled for different intracellular structures. Upper small pictures: Nuclei (DAPI channel, DAPI, 12 ms), Focal adhesions (425 channel, anti-vinculin, 90 ms), Lipid droplets (488 channel, anti-ADRP, 50 ms), Golgi (Cy3 channel, anti-syntaxin 5, 10 ms), F-actin cytoskeleton (594 channel, phalloidin, 17 ms), and Lyzosomes (PerCP channel, anti-LAMP 1, 46 ms). Scale bar, 40 μm. Objective x63/1.4 Oil DIC. Lower large picture: Merged image of all channels. Scale bar, 20 μm.

To verify the performance of the FDMM setup, we multi-immunolabeled cultured cells (NIH fibroblasts, Fig. 2) and tissue sections from a mouse atherosclerotic plaque (S3 Fig.). We found that the FDMM setup worked well and did not require any new operative steps compared with standard multicolor microscopy. All fluorochrome channels could be used simultaneously on one tissue section without bleed-through issues. This was true at both the subcellular level (cultured cells) and the multicellular level (tissue sections). Thus, the FDMM setup is easy to apply to a standard fluorescence microscope and it generates specific signals from each fluorochrome.

### The FDMM setup facilitates studies that require many cell markers

**Fig 3:**
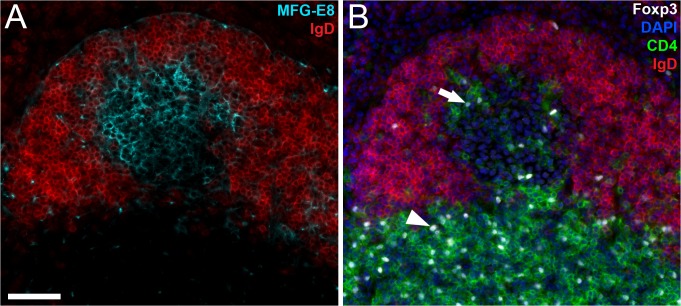
Detection of regulatory T cells in germinal centers using FDMM. Mice were immunized i.p. with a mixture of polyI:C and ovalbumin to induce formation of germinal centers. Ten days later the spleens were harvested, sectioned and multi-immunolabeled with antibodies against IgD-expressing B cells (anti-IgD, red), follicular dendritic cells (anti-MFG-E8, cyan), CD4^+^ T cells (anti-CD4, green), the transcription factor Foxp3 (anti-Foxp3, white), B cells (anti-B220, not shown) and nuclei (DAPI, blue). *(A)* Detection of a germinal center. The picture shows the expression of IgD (red, negative marker) and MGF-E8 (cyan, positive marker). *(B)* Detection of regulatory T cells (CD4^+^Foxp3^+^) in a germinal center (arrow) and outside a germinal center (arrowhead). All channels are presented individually in S4 Fig. Scale bar, 50 μm.

Increasing the number of fluorescence channels facilitates microscopic analyses that require many cell markers to define the tissue structure and/or cellular subtypes. A typical example in immunology is detection of specific cell types in germinal centers, i.e. the structures in lymph nodes and spleen where clonal expansion of high affinity memory B cells and long-lived plasma cells take place [4]. In the present study, we used FDMM to detect regulatory T cells in germinal centers, so-called follicular regulatory T cells (T_FR_) [10], in mouse spleen following immunization with antigen + adjuvant. All individual fluorescence channels for this immunolabeling experiment are shown in S4 Fig. To detect the presence of a germinal center, we used antibodies against IgD (negative marker) and MFG-E8 (positive marker, Fig. 3A) [4]. To detect regulatory T cells, we used antibodies against CD4 and Foxp3 [2]. Cell nuclei were stained with DAPI. Together, these markers revealed regulatory T cells (CD4^+^Foxp3^+^) within germinal centers in addition to those outside germinal centers (Fig. 3B). Only five of the six channels were used to produce the images shown in Fig. 3, and we used the sixth channel to visualize B cells (shown in S4 Fig.). This channel could of course be used for any protein of interest or a cell tracker dye.

**Fig 4:**
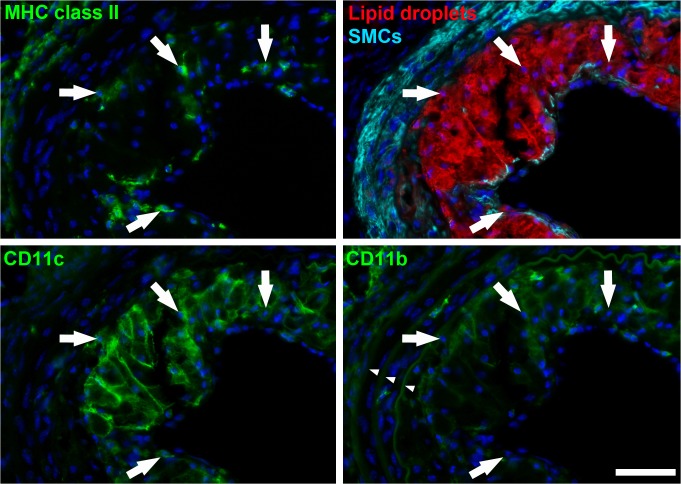
Detection of lipid-loaded dendritic cells in an atherosclerotic lesion using FDMM. Atherosclerotic lesions were induced in the carotid artery of LDLr^−/−^ mice. The carotid arteries were harvested, sectioned and multi-immunolabeled with antibodies against lipid droplets (anti-perilipin 2, red), smooth muscle cells (anti-SM α-actin, cyan), antigen presenting cells (anti-MHC class II, green, upper left), CD11c (anti-CD11c, green, lower left), and CD11b (anti-CD11b, green, lower right). Nuclei were stained with DAPI (blue). Arrows depict cells that are positive for MHC class II, CD11c and perilipin 2, and negative/dim for CD11b. The small arrowheads indicate arterial elastin fibers (laminae), which autofluoresce in the 425 channel. All six channels are presented individually in S5 Fig. Scale bar, 100 μm.

In another example, we used the FDMM setup to visualize mononuclear phagocytes in atherosclerotic lesions. It is well known that macrophages accumulate lipids to become foam cells, but recent evidence suggests that dendritic cells can also accumulate lipids [11]. To distinguish dendritic cells from macrophages, we immunolabeled tissue against CD11c, MHC class II and CD11b [3]; to detect intracellular lipids, we immunolabeled against the lipid droplet protein perilipin 2; and to visualize the vascular morphology, we immunolabeled smooth muscle cells (anti-smooth muscle α-actin). All individual fluorescence channels are shown in S5 Fig. We found several MHC class II^+^ cells that had accumulated lipids (perilipin 2^+^, Fig. 4, upper panels). Many of these cells were CD11c^+^, CD11b^−/dim^ (Fig. 4, lower panels), suggesting that they are dendritic cells rather than macrophages [3,11,12], although more cell markers are preferentially needed to confirm the cell type. Taken together, these examples demonstrate the utility of additional fluorescence channels in histological analyses that involve multiple cell markers.

### The FDMM setup can be used for multicolor antibody arrays

**Fig 5:**
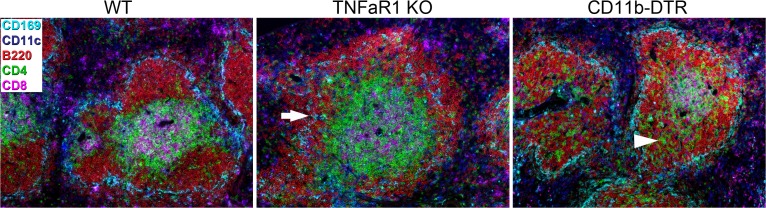
Multicolor antibody array for mouse spleen using FDMM. Tissue sections from a wild type spleen (WT), a TNFα-receptor 1 knockout spleen (TNFaR1 KO), and a spleen from a CD11b-DTR mouse (CD11b-DTR) were multi-immunolabeled with antibodies against marginal zone macrophages (anti-CD169, cyan), dendritic cells (anti-CD11c, blue), B cells (anti-B220, red), CD4^+^ T cells (anti-CD4, green), and CD8^+^ T cells (anti-CD8, magenta). Nuclei were stained with DAPI (not shown). Arrow in TNFα-receptor 1 knockout spleen indicates weak marginal zone structure (cyan). Arrowhead in CD11b-DTR spleen indicates CD4^+^ T cells (green) within the B cell follicles. All six channels are presented individually in S6 Fig. Scale bar, 100 μm.

Another beneficial use of FDMM is multicolor antibody arrays for tissue characterizations. As a specific example, we designed an array that labels the main cell types in the mouse spleen within one tissue section (B cells, CD4^+^ T cells, CD8^+^ T cells, dendritic cells, marginal zone macrophages, and nuclei). The antibody array was used on spleen sections from a wild type and a TNFαR1 knockout mouse [13] and from a CD11b-DTR mouse treated with diphtheria toxin [14]. All individual fluorescence channels for this immunolabeling experiment are shown in S6 Fig. The array readily revealed the known spleen phenotype of the TNFαR1 knockout mouse, with partly dislocated B cells as a result of a weak marginal zone structure (Fig. 5) [13]. Furthermore, we also detected an intriguing spleen phenotype in the CD11b-DTR mouse. Although this spleen had well-defined lymphoid follicles, a substantial number of CD4^+^ T cells had populated the B cell follicles, while the CD8^+^ T cells were still located at the core of the follicular T cell area (Fig. 5). These data conclusively demonstrate that different structural phenotypes of a tissue can easily be detected using an antibody array that displays the relative location of its main cell types.

## Discussion

Although many bright and photostable fluorochromes have been developed in the past 20 years [15–17], the filter set arrangements have basically remained the same, and often span a much broader wavelength interval than necessary to receive strong signals. Here we demonstrate that the number of channels in multicolor microscopy can be significantly increased by using FDMM. FDMM is based on condensed filter sets that are more specific for each fluorochrome and occupy smaller portions of the light spectrum. Consequently, the condensed filter sets in combination with a selected panel of fluorochromes allow a more efficient use of the available light spectrum compared with current technology. In the present study, we show an FDMM setup of six independent fluorescence channels within the visible and near infrared light spectra. Five of these fluorochromes are visible by eye and can be analyzed directly under the microscope, and all of them can be activated by an ordinary mercury lamp or a metal halide lamp (X-cite, data not shown).

The FDMM setup includes four of the most commonly used fluorochromes for histology, namely DAPI, 488 dyes, Cy3, and 594 dyes. Besides these standard fluorochromes, we included Atto425 in the spectrum between DAPI and 488 dyes. There are only a few available fluorochromes with suitable excitation/emission spectra at these wavelengths, and most of them are dim or affected by photobleaching. However, Atto425 is bright and photostable, and its spectra fitted well into the FDMM setup (S1 Fig.). For the near infrared spectrum, we chose to use PerCP-based fluorochromes [18]. These fluorochromes have large Stoke shifts (i.e. a large shift of emission spectra relative to excitation spectra) and can be activated at the blue-yellow spectra (400–550 nm), where the illumination from the mercury lamp is strong [19]. Although PerCP is affected by photobleaching, the tandem dyes PerCP-Cy5.5 and PerCP-eFluor710 are bright and photostable, and work well for histological immunofluorescence (our observations).

Increasing the number of channels opens up new potential uses for multicolor microscopy and updates the technology in line with progress in biomedical science. A striking advance in recent years has been the discovery and use of a rapidly increasing number of marker proteins to classify functionally distinct cell populations and diseased tissues [1–3,10,20–23]. This trend causes a situation where regular multicolor microscopy does not have enough channels to visualize a certain cell type and its cellular context. On top of this, there is often an additional channel required for a certain protein of interest or a cell tracker dye. In the present study, we show two typical examples that require many cell markers: first, detection of regulatory T cells in germinal centers (T_FR_) [10], and second, detecting lipid-loaded dendritic cells in atherosclerotic lesions [11]. Although several cell markers were used for detection of certain cell types, the FDMM setup had channels available to visualize the tissue context.

Additional fluorescence channels also benefit the use of multicolor antibody arrays. Development of pre-designed antibody arrays for all common tissues, signaling pathways, cell subtypes, etc. would greatly enhance the speed and performance of histological analyses. The power of the multicolor array approach lies in the fact that each extra channel increases the biological information dramatically. For example, six double-stainings would be required to obtain all the localization information for 4 different proteins (3+2+1), while 15 double-stainings would be required to obtain this information for 6 different proteins (5+4+3+2+1). In the present study, we designed an antibody array with five antibodies + nuclear dye for mouse spleen. The array showed that different spleen phenotypes could easily be detected when the relative location of different cell types was revealed in one tissue section. Importantly, these phenotypes are not obvious when looking at individual images (see S6 Fig.), and become clear only when they are applied on top of each other (Fig. 5). These results demonstrate the potential power of multicolor antibody arrays to gain histological insight. The multicolor array in the present study was based on six channels illuminated by a mercury lamp. Using a light source that also emits strong light in the near infrared/infrared spectra, such as a xenon lamp or light-emitting diodes (LEDs) [19], would allow an additional channel to be used and thus further advance the multicolor array approach.

In conclusion, we have developed an enhanced multicolor setup that allows a more efficient approach to analyze protein expression in tissue sections and cultured cells. The FDMM setup includes the fluorochromes most commonly used for histology, and the setup does not change any operative procedures for the user compared with regular fluorescence microscopy. Together these features make the FDMM setup easy to adapt and easy to use.

## Methods

### Microscope

The condensed filter sets are defined in S1 Table. Pictures were taken under a regular fluorescence microscope system from Carl Zeiss Microscopy (Axioplan 2 microscope, AxioCam MRm Camera, Axiovision 4.1 software, ebq 100 isolated mercury lamp), with apochromat objectives (x10/0.45, x20/0.75, x63/1.4 Oil DIC). Exposure times are indicated in figure legends.

### Mice and tissue preparation

Carotid arteries with intimal hyperplasia [24] were obtained from C57Bl/6 wild type mice. Spleens were obtained from C57Bl/6 wild type mice (n = 2), TNFα-receptor1−/− mice (n = 2) [13] and a CD11c-DTR mouse (n = 1) [14] that was treated with three diphtheria toxin injections (15 ng/kg, i.p., day 0, 3 and 6). Spleens with germinal centers were obtained from C57Bl/6 wild type mice that had been immunized intraperitoneally with 100 micrograms polyI:C (InvivoGen) and 300 micrograms of Ovalbumin (Sigma), ten days before tissue harvest (n = 2). Carotid arteries with atherosclerotic lesions were obtained from LDLr−/− mice that had been subjected to a carotid injury [24] and put on western diet for 5 weeks (n = 2). All animal procedures were approved by the Gothenburg animal ethics committee (permit numbers 290–2012, 309–2012, 34–2014) at the University of Gothenburg. All tissues were snap frozen in liquid nitrogen and cryo-sectioned in 10 μm thick sections. The sections were air dried for 2 h and stored at-20°C. For immunolabeling, sections were fixed for 5 min with 2% formaldehyde, permeabilized with 0.1% Triton-X for 4 min, treated with Avidin/Biotin Blocking kit (Vector Laboratories), and finally incubated with 1% BSA in PBS for 15 min, before adding primary antibodies. PBS was used for all washes throughout the immunolabeling procedures except for the last wash before mounting, which was done in deionized water.

### Immunolabeling of endothelium

The endothelium of mouse carotid arteries with intimal hyperplasia (Fig. 1C and S2 Fig.) was immunolabeled with biotinylated goat anti-mouse CD31 antibodies at 4°C overnight (1:200, R&D Systems). The samples were then incubated for 45 min with streptavidin conjugated with different fluorochromes: Atto425 (1:200, ATTO-Tec), CF488 (1:200, Biotium), Cy3 (1:200, Biolegend), Dylight594 (1:200, Biolegend), and PerCP-Cy5.5 (1:100, Biolegend).

### Immunolabeling of atherosclerotic lesion

Arterial tissue sections (S3 Fig.) were incubated overnight at 4°C with rat anti-mouse CD18 (1:50, clone C71/16, Cedarlane), Cy3-conjugated mouse-anti human α-actin (1:15000, clone 1A4, Sigma-Aldrich), goat anti-mouse/human ApoB (1:100, R&D systems), and rabbit anti-Ki67 (1:100, clone SP6, GeneTex). The sections were washed and incubated with F(ab)2 AF594-conjugated monovalent donkey anti-goat (1:200, Jackson ImmunoResearch,) and biotinylated donkey anti-rabbit (1:200, Jackson ImmunoResearch) at room temperature for 45 min, followed by Atto425-conjugated streptavidin (1:200, ATTO-TEC) and AF488-conjugated goat anti-mouse CD31 (1:150, R&D systems) at room temperature for 45 min. Nuclei were stained with DAPI (2 μg/ml for 4 min) and the slides were mounted with ProlongGold mounting media (Life Technologies).

### Immunostaining of cultured cells

NIH cells (Fig. 2) were seeded onto cover slips placed in 24-well plates. After 24 h, the medium was changed to new medium containing 360 μM oleic acid. After 20 h, the cells were fixed with 2% formaldehyde for 10 min and stored at 4°C in PBS. For immunolabeling, the cells were treated with Avidin/Biotin Blocking kit (Vector Laboratories), and 1% BSA in PBS for 20 min. The cells were then incubated 2h at room temperature with mouse anti-vinculin (1:100, clone SPM227, Abcam), followed by 30 min biotinylated monovalent donkey anti-mouse IgG (1:200, Jackson ImmunoResearch) and 30 min with Atto425-conjugated streptavidin (1:200, ATTO-TEC). The cells were then incubated for 2h at room temperature with guinea pig anti-ADRP (1:400, Fitzgerald), rabbit anti-mouse syntaxin 5 (1:150, Synaptic System) and rat anti-mouse LAMP1 (1:100, clone 1D4B, Abcam) followed by 30 min incubation with goat anti-guinea pig AF488 (1:400, Life Technologies), goat anti-rabbit Cy3 (1:250, Jackson ImmunoResearch), mouse anti-rat PerCP-eFluor710 (1:100, Affymetrix) and phalloidin-Atto594 (5 μL/mL, Sigma-Aldrich); in the last 5 min, 3 drops of DAPI (2 μg/mL) was added to each well. All incubation steps contained 0.1% saponin and all washing steps contained 0.05% saponin for permeabilization. The cover slips were mounted onto microscope slides with Prolong Gold antifade reagent (Life Technologies).

### Immunolabeling of regulatory T cells in germinal centers

Spleen tissue sections with germinal centers (Fig. 3 and S4 Fig.) were incubated for 2h at 20°C with rat anti-mouse Foxp3 (1:100, Clone FJK-16s, Affymetrix) and hamster anti-MFGE8 (1:100, clone 18A2-G10, MBL) followed by 45 min with F(ab)2 AF594-conjugated donkey anti-rat IgG (1:400, Jackson ImmunoResearch) and Cy3-conjugated goat anti-Armenian hamster IgG (1:400, Jackson ImmunoResearch). The sections were then blocked 15 min with nonspecific rat IgG2a (1:30, Clone R35-95, BD Biosciences) and incubated 60 min with AF488-conjugated rat anti-human/mouse B220 (1:100, clone RA3-6B2, Affymetrix), biotinylated rat anti-mouse CD4 (1:100, clone RM4-5, Affymetrix) and PerCP-eFluor710-conjugated rat anti-mouse IgD (1:100, clone 11–26c, Biolegend). The sections were then incubated 30 min with Atto425-conjugated streptavidin (1:300, ATTO-TEC). Nuclei were stained with DAPI (2 μg/ml for 4 min) and the slides were mounted with ProlongGold mounting media (Life Technologies).

### Immunolabeling of lipid-loaded dendritic cells in atherosclerotic lesions

Arterial tissue sections (Fig. 4) were incubated with rat anti-mouse MHC class II (1:300, Clone 2G9, Affymetrix) and Guinea pig anti-perilipin 2 (1:100, perilipin 2/adipophilin cat# 20R-AP002, **Fitzgerald Industries International**) for 2h at 20°C, followed by F(ab)2 AF594-conjugated donkey anti-rat IgG (1:400, Jackson ImmunoResearch) and AF488-conjugated goat anti-Guinea pig IgG (1:400, Invitrogen). The sections were blocked 15 min with nonspecific rat IgG2b (1:30, Clone A95–1, BD Biosciences) and incubated with PerCP-Cy5.5-conjugated hamster anti-mouse CD11c (1:50, clone N418, Affymetrix), biotinylated rat anti-mouse CD11b (1:200, clone M1/70, Affymetrix), and Cy3-conjugated mouse anti-smooth muscle α-actin (1:3000, clone 1A4, Sigma). The sections were then incubated 30 min with Atto425-conjugated streptavidin (1:300, ATTO-TEC). Nuclei were stained with DAPI (2 μg/ml for 4 min) and the slides were mounted with ProlongGold mounting media (Life Technologies).

### Multicolor antibody array for spleen

Spleen tissue sections (Fig. 5) were incubated overnight at 4°C with rat anti-mouse CD169 (1:100, Clone 3D6.112, AbD Serotec) and hamster anti-mouse CD11c (1:100, clone N418, Affymetrix) followed by 45 min with F(ab)2 AF594-conjugated donkey anti-rat IgG (1:400, Jackson ImmunoResearch) and Cy3-conjugated goat anti-Armenian hamster IgG (1:300, Jackson ImmunoResearch). The sections were then blocked 15 min with nonspecific rat IgG2a (1:30, Clone R35-95, BD Biosciences) and incubated 60 min with AF488-conjugated rat anti-human/mouse B220 (1:100, clone RA3-6B2, Affymetrix), biotinylated rat anti-mouse CD4 (1:100, clone RM4-5, Affymetrix) and PerCP-Cy5.5 conjugated anti-mouse CD8a (1:50, clone 53–6.7, Affymetrix). The sections were then incubated 30 min with Atto425-conjugated streptavidin (1:300, ATTO-TEC). Nuclei were stained with DAPI (2 μg/ml for 4 min) and the slides were mounted with ProlongGold mounting media (Life Technologies).

## Supporting Information

S1 Fig The filter set arrangements for each channel of the FDMM setup. Excitation spectra (blue lines) and emission spectra (red lines) of representative fluorochromes that fit in the FDMM setup, and corresponding light filter intervals for excitation (blue rectangles) and emission (red rectangles). Vertical black line indicates the beam splitter. The gray rectangles depict the excitation and emission intervals of representative standard filter sets.
(TIF)

S2 Fig No bleed-through signals in the condensed filter sets when exposure times were increased. Images of the same immunolabeled tissue sections as in Fig. 1C taken with five time’s longer exposure times for each filter set channel. Objective x20/0.75. Exposure times: DAPI channel, 55 ms; 425 channel, 150 ms; 488 channel, 315 ms; Cy3 channel, 100 ms; 594 channel, 270 ms; PerCP channel, 85 ms.
(TIF)

S3 Fig Six channel FDMM for histological analysis of an atherosclerotic lesion. Tissue section from a mouse atherosclerotic plaque, immunolabeled for different cell types, lipoproteins and proliferating nuclei. Upper small pictures: Nuclei (DAPI channel, DAPI, 18 ms), Proliferation (425 channel, anti-Ki67, 300 ms), Endothelial cells (488 channel, anti-CD31, 536 ms), Smooth muscle cells (Cy3 channel, anti-α-actin, 349 ms), Lipoproteins (594 channel, anti-apoB, 450 ms), Leukocytes (PerCP channel, anti-CD18, 671 ms). Scale bar, 200 μm. Objective x10/0.45 Lower large picture: Merged image of all channels. Scale bar, 100 μm.
(TIF)

S4 Fig Detection of regulatory T cells in germinal centers using FDMM—individual fluorescence channels. Mice were immunized to induce formation of germinal centers. Ten days later the spleens were harvested, sectioned and multi-immunolabeled with antibodies against T cells, B cells, follicular dendritic cells (FDC), regulatory T cells (T regs) and IgD-expressing B cells. Nuclei were stained with DAPI. The texts in the figure indicate what structures that have been immunolabeled, followed by the antigens and the fluorochromes within brackets. Objective x20/0.75. Exposure times: DAPI channel, 46 ms; 425 channel, 277 ms; 488 channel, 660 ms; Cy3 channel, 82 ms; 594 channel, 1350 ms; PerCP channel, 341 ms. Scale bar, 100 μm. Merged images are shown in Fig. 3.
(TIF)

S5 Fig Detection of lipid-loaded dendritic cells in an atherosclerotic lesion using FDMM—individual fluorescence channels. Atherosclerotic lesions were induced in the carotid artery of LDLr^−/−^ mice. The carotid arteries was harvested, sectioned and multi-immunolabeled with antibodies against CD11b, lipid droplets, smooth muscle cells (SMC), antigen presenting cells, and CD11c. Nuclei were stained with DAPI. The texts in the figure indicate what structures that have been immunolabeled, followed by the antigens and the fluorochromes within brackets. Objective x20/0.75. Exposure times: DAPI channel, 18 ms; 425 channel, 456 ms; 488 channel, 198 ms; Cy3 channel, 84 ms; 594 channel, 982 ms; PerCP channel, 715 ms. Scale bar, 100 μm. Merged images are shown in Fig. 4.
(TIF)

S6 Fig Multicolor antibody array for mouse spleen using FDMM—individual fluorescence channels. Tissue sections from a wild type spleen (WT), a TNFα-receptor 1 knockout spleen (TNFaR1 KO), and a spleen from a CD11b-DTR mouse (CD11b-DTR) were multi-immunolabeled with antibodies against CD4^+^ T cells, B cells, dendritic cells, marginal zone macrophages, and CD8^+^ T cells. Nuclei were stained with DAPI. Texts on the left side indicate what structures that have been immunolabeled, followed by the antigens and the fluorochromes within brackets. Objective x20/0.75. Exposure times: DAPI channel, 62 ms; 425 channel, 796 ms; 488 channel, 2500 ms; Cy3 channel, 1201 ms; 594 channel, 886 ms; PerCP channel, 3469 ms. Scale bar, 100 μm. Merged images are shown in Fig. 5.
(TIF)

S1 Table Filter set arrangements. (DOCX)

S2 Table Calculated collected fraction of total emission signal, signal-to-noise ratios, signal-to-bleed-through ratios of the FDMM filter sets. (DOCX)

S3 Table Calculated collected fraction of total emission signal, signal-to-noise ratios, signal-to-bleed-through ratios on standard filter sets from Chroma Technology Corp. (DOCX)

S4 Table Calculated collected fraction of total emission signal, signal-to-noise ratios, signal-to-bleed-through ratios on standard filter sets from Carl Zeiss Microscopy. (DOCX)
