# Supplementary figures and images for: Filter-Dense Multicolor Microscopy
Source: PLoS One. 2015 Mar 4;10(3):e0119499. doi: 10.1371/journal.pone.0119499 (PMC4349739; doi:10.1371/journal.pone.0119499)

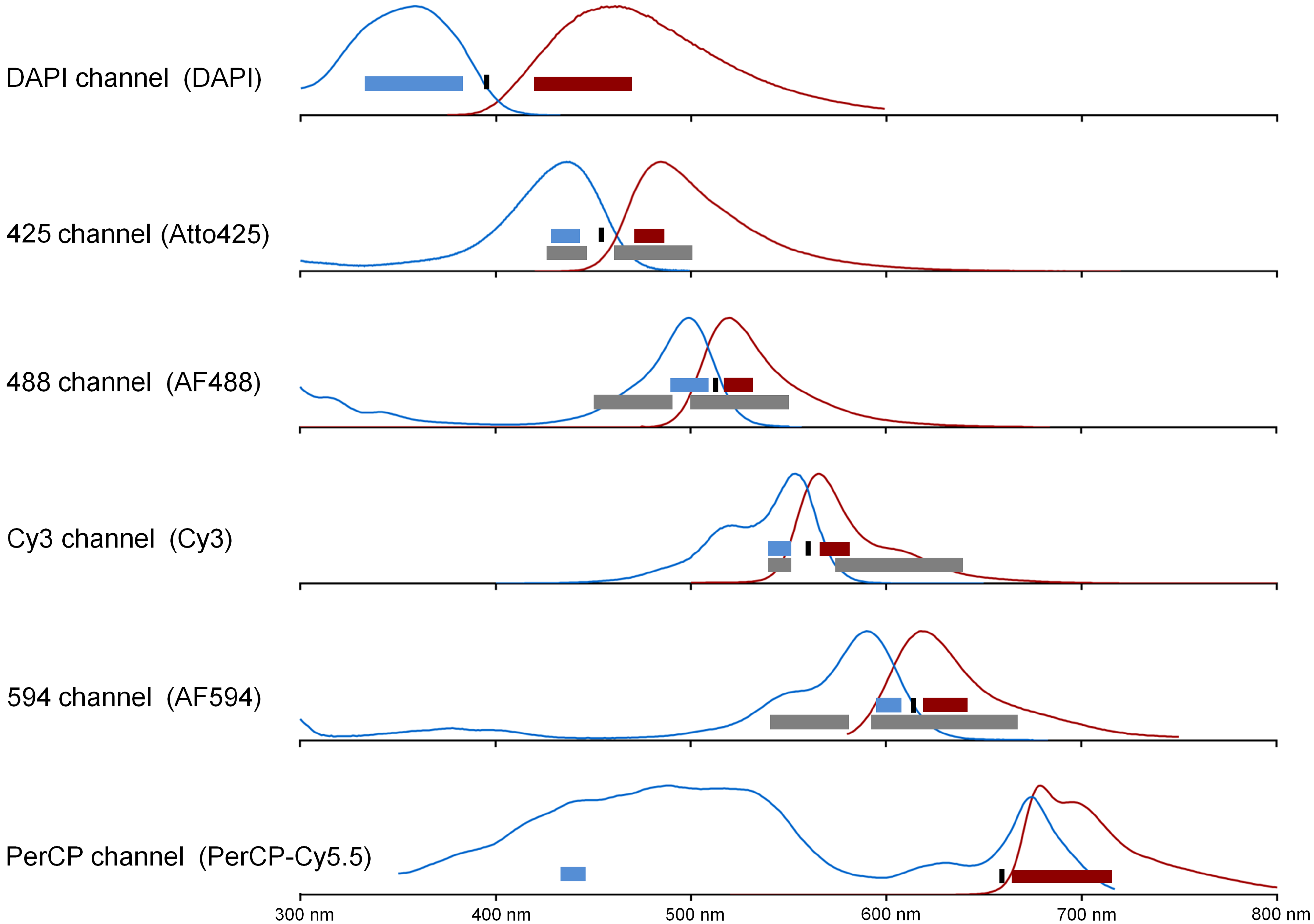

Supplement: S1 Fig — Excitation spectra (blue lines) and emission spectra (red lines) of representative fluorochromes that fit in the FDMM setup, and corresponding light filter intervals for excitation (blue rectangles) and emission (red rectangles). Vertical black line indicates the beam splitter. The gray rectangles depict the excitation and emission intervals of representative standard filter sets. (TIF) [file pone.0119499.s001.tif]

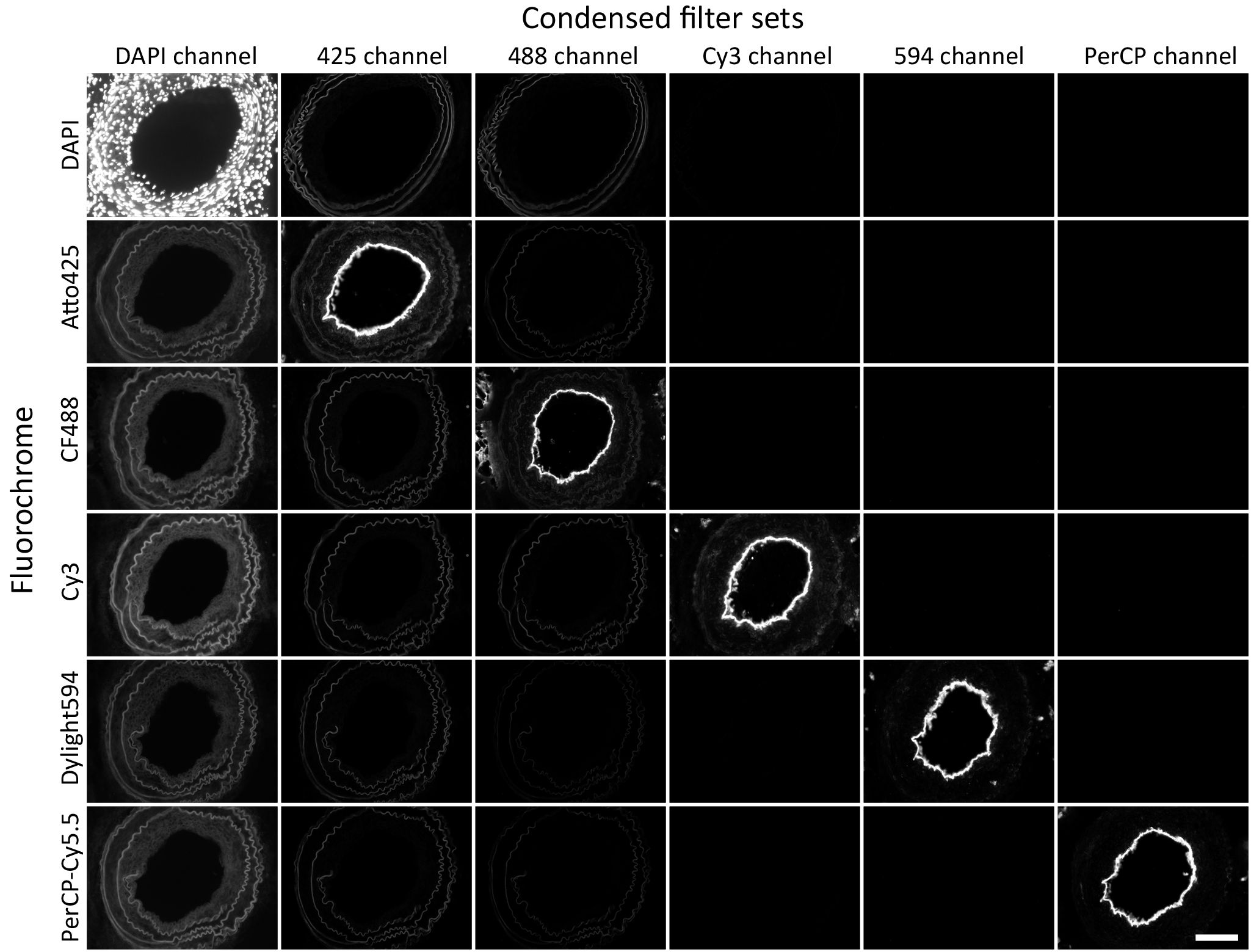

Supplement: S2 Fig — Images of the same immunolabeled tissue sections as in Fig. 1C taken with five time’s longer exposure times for each filter set channel. Objective x20/0.75. Exposure times: DAPI channel, 55 ms; 425 channel, 150 ms; 488 channel, 315 ms; Cy3 channel, 100 ms; 594 channel, 270 ms; PerCP channel, 85 ms. (TIF) [file pone.0119499.s002.tif]

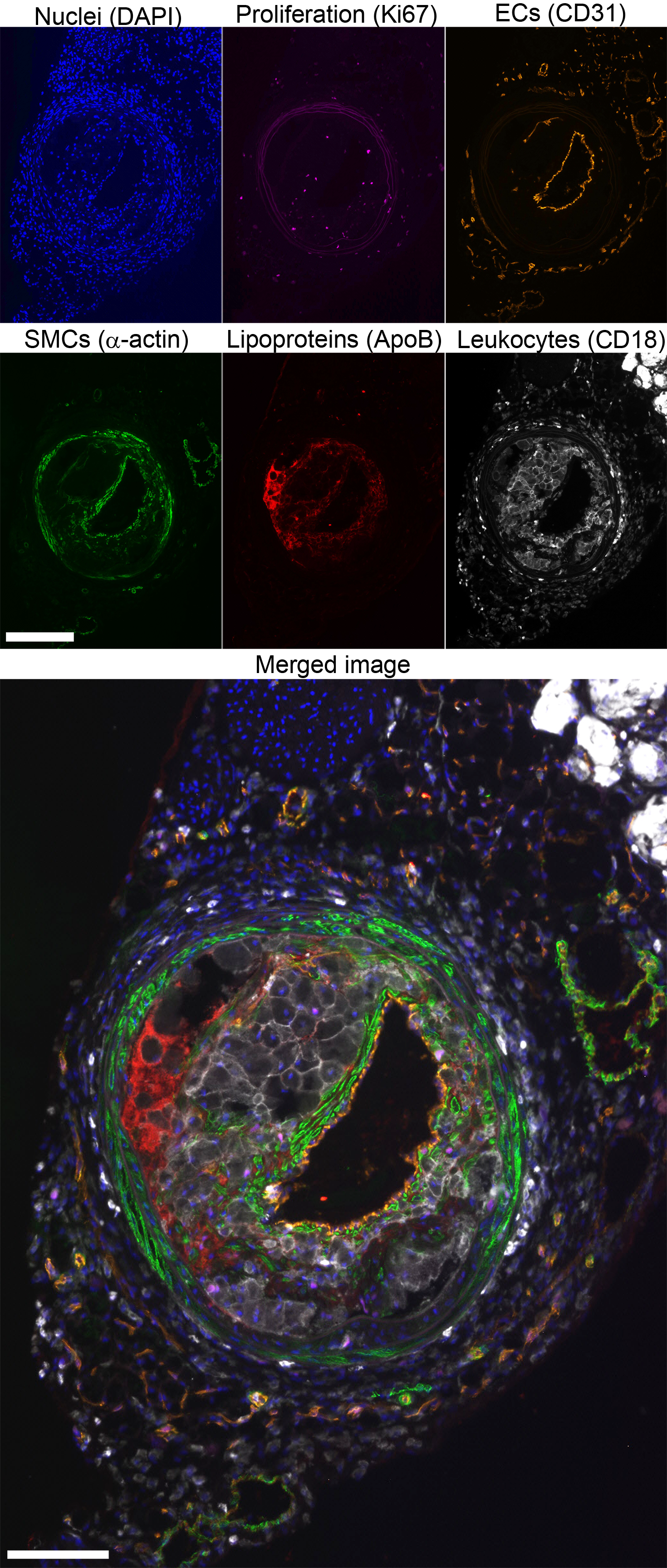

Supplement: S3 Fig — Tissue section from a mouse atherosclerotic plaque, immunolabeled for different cell types, lipoproteins and proliferating nuclei. Upper small pictures: Nuclei (DAPI channel, DAPI, 18 ms), Proliferation (425 channel, anti-Ki67, 300 ms), Endothelial cells (488 channel, anti-CD31, 536 ms), Smooth muscle cells (Cy3 channel, anti-α-actin, 349 ms), Lipoproteins (594 channel, anti-apoB, 450 ms), Leukocytes (PerCP channel, anti-CD18, 671 ms). Scale bar, 200 μm. Objective x10/0.45 Lower large picture: Merged image of all channels. Scale bar, 100 μm. (TIF) [file pone.0119499.s003.tif]

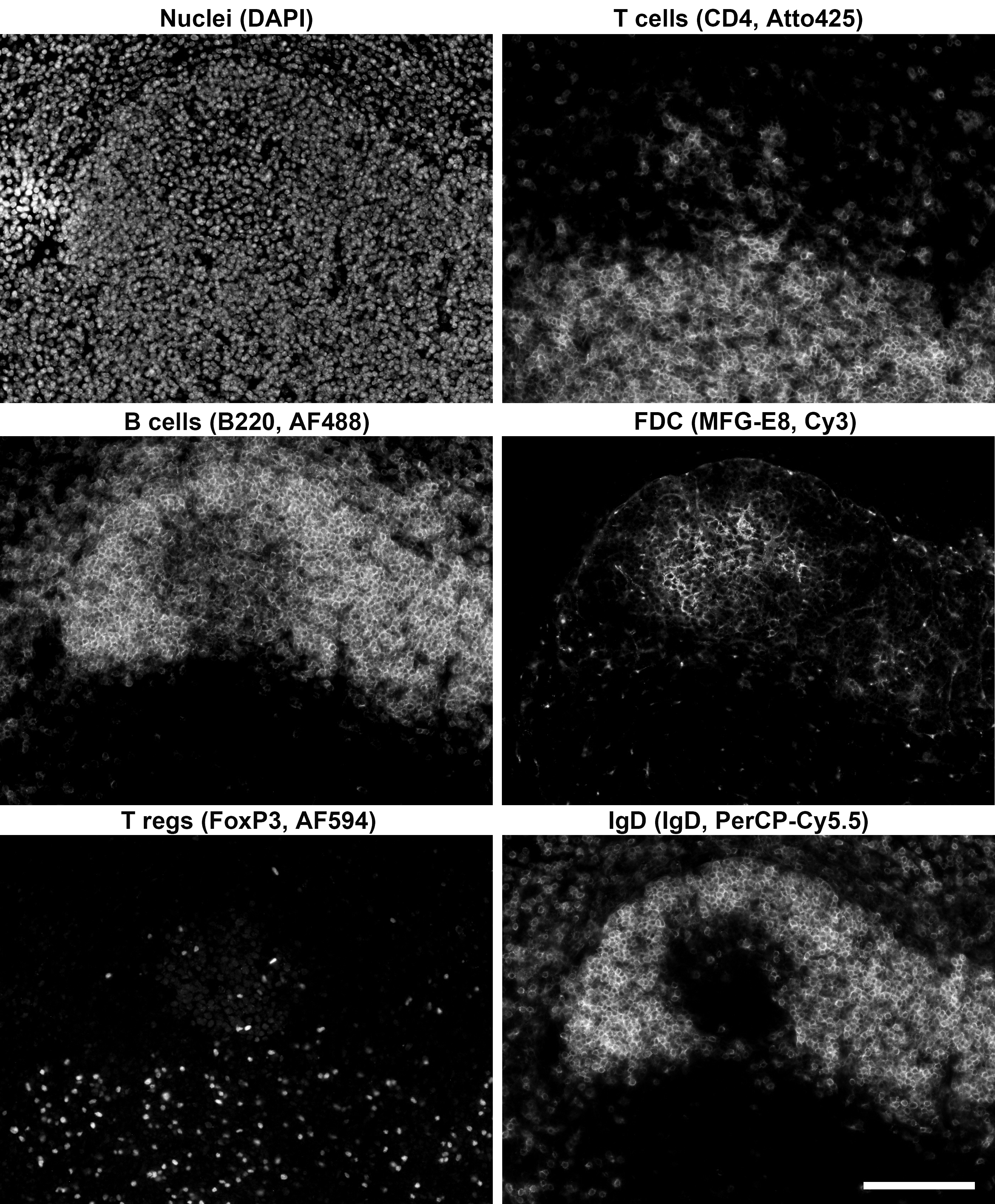

Supplement: S4 Fig — Mice were immunized to induce formation of germinal centers. Ten days later the spleens were harvested, sectioned and multi-immunolabeled with antibodies against T cells, B cells, follicular dendritic cells (FDC), regulatory T cells (T regs) and IgD-expressing B cells. Nuclei were stained with DAPI. The texts in the figure indicate what structures that have been immunolabeled, followed by the antigens and the fluorochromes within brackets. Objective x20/0.75. Exposure times: DAPI channel, 46 ms; 425 channel, 277 ms; 488 channel, 660 ms; Cy3 channel, 82 ms; 594 channel, 1350 ms; PerCP channel, 341 ms. Scale bar, 100 μm. Merged images are shown in Fig. 3. (TIF) [file pone.0119499.s004.tif]

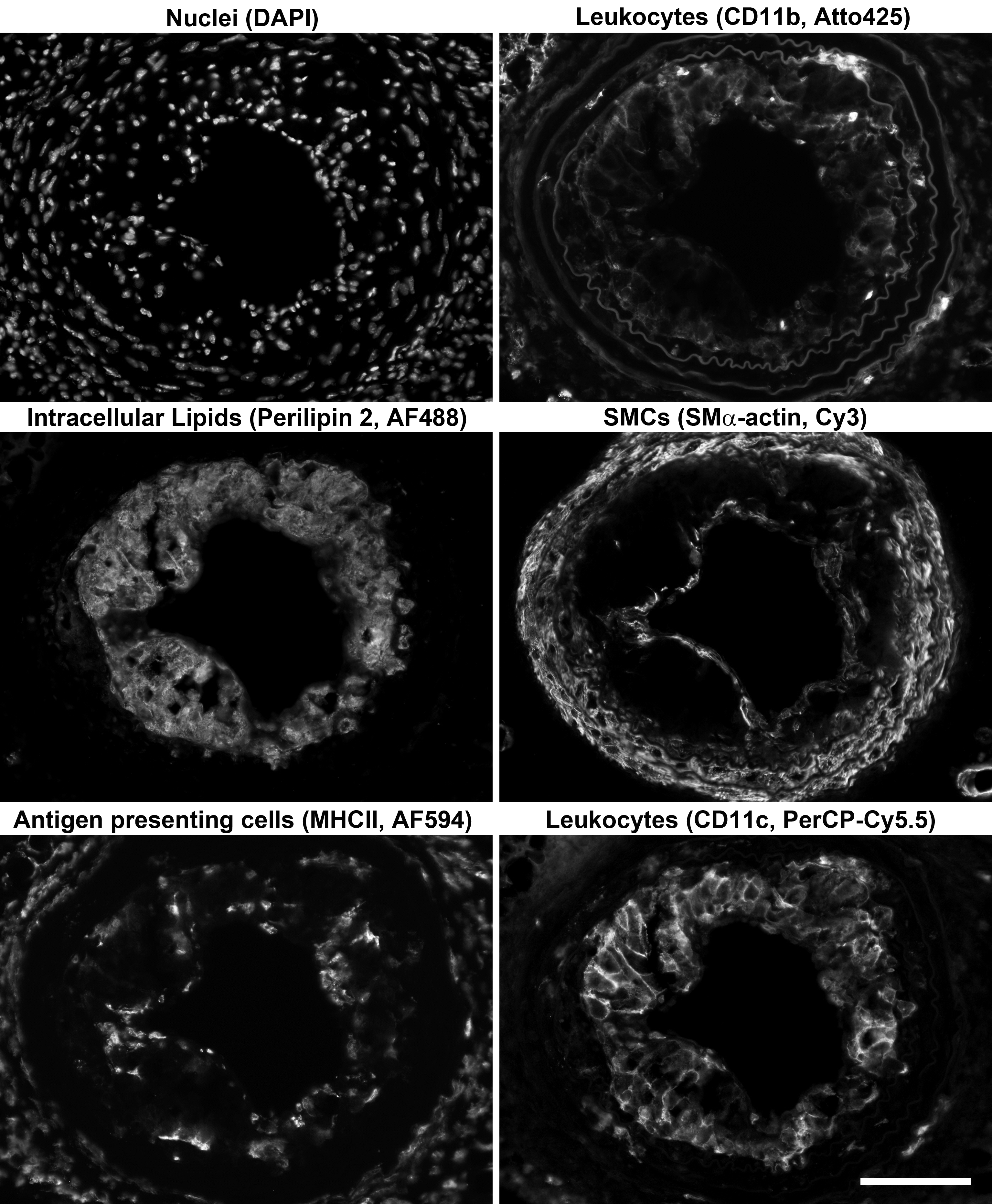

Supplement: S5 Fig — Atherosclerotic lesions were induced in the carotid artery of LDLr−/− mice. The carotid arteries was harvested, sectioned and multi-immunolabeled with antibodies against CD11b, lipid droplets, smooth muscle cells (SMC), antigen presenting cells, and CD11c. Nuclei were stained with DAPI. The texts in the figure indicate what structures that have been immunolabeled, followed by the antigens and the fluorochromes within brackets. Objective x20/0.75. Exposure times: DAPI channel, 18 ms; 425 channel, 456 ms; 488 channel, 198 ms; Cy3 channel, 84 ms; 594 channel, 982 ms; PerCP channel, 715 ms. Scale bar, 100 μm. Merged images are shown in Fig. 4. (TIF) [file pone.0119499.s005.tif]

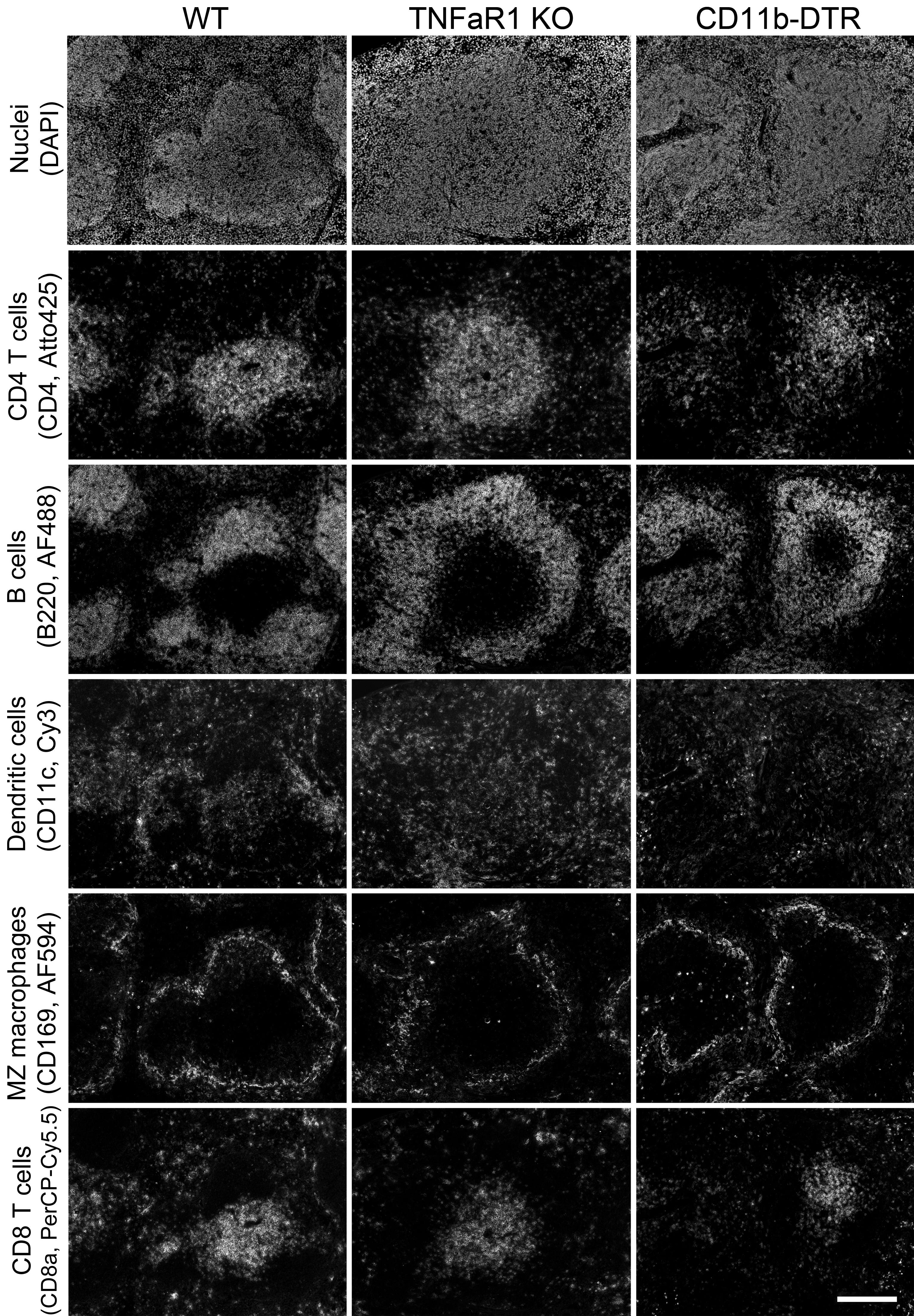

Supplement: S6 Fig — Tissue sections from a wild type spleen (WT), a TNFα-receptor 1 knockout spleen (TNFaR1 KO), and a spleen from a CD11b-DTR mouse (CD11b-DTR) were multi-immunolabeled with antibodies against CD4+ T cells, B cells, dendritic cells, marginal zone macrophages, and CD8+ T cells. Nuclei were stained with DAPI. Texts on the left side indicate what structures that have been immunolabeled, followed by the antigens and the fluorochromes within brackets. Objective x20/0.75. Exposure times: DAPI channel, 62 ms; 425 channel, 796 ms; 488 channel, 2500 ms; Cy3 channel, 1201 ms; 594 channel, 886 ms; PerCP channel, 3469 ms. Scale bar, 100 μm. Merged images are shown in Fig. 5. (TIF) [file pone.0119499.s006.tif]
