# Supplementary material for: Filter-Dense Multicolor Microscopy
Source: PLoS One. 2015 Mar 4;10(3):e0119499. doi: 10.1371/journal.pone.0119499 (PMC4349739; doi:10.1371/journal.pone.0119499)
Supplement: S1 Table — (DOCX) [file pone.0119499.s007.docx]

| **Table S1**. Filter set arrangements | | | |
| --- | --- | --- | --- |
| Filter set | Filters/Mirrors | Description | Manufacturer |
| DAPI | Excitation filter | 350/50 nm | Carl Zeiss Microscopy |
|  | Beam splitter | 395 LP | Carl Zeiss Microscopy |
|  | Emission filter | 445/50 nm | Carl Zeiss Microscopy |
| 425 | Excitation filter | 442/10 nm | Edmund Optics |
|  | Beam splitter | 455 LP | Carl Zeiss Microscopy |
|  | Emission filter | 480/10 nm | Edmund Optics |
| 488 | Excitation filter | 500/20 nm | Carl Zeiss Microscopy |
|  | Beam splitter | 515 LP | Carl Zeiss Microscopy |
|  | Emission filter | 525/15 nm | Edmund Optics |
| Cy3 | Excitation filter | 546/12 nm | Carl Zeiss Microscopy |
|  | Beam splitter | 560 LP | Carl Zeiss Microscopy |
|  | Emission filter | 575/15 nm | Semrock |
| 594 | Excitation filter | 602/13 nm | Chroma Technology |
|  | Beam splitter | 615 LP | Chroma Technology |
|  | Emission filter | 631/23 nm | Chroma Technology |
| PerCP | Excitation filter | 442/10 nm | Edmund Optics |
|  | Beam splitter | 660 LP | Carl Zeiss Microscopy |
|  | Emission filter | 690/50 nm | Carl Zeiss Microscopy |
