# Supplementary material for: Filter-Dense Multicolor Microscopy
Source: PLoS One. 2015 Mar 4;10(3):e0119499. doi: 10.1371/journal.pone.0119499 (PMC4349739; doi:10.1371/journal.pone.0119499)
Supplement: S2 Table — (DOCX) [file pone.0119499.s008.docx]

| **Table S2.** Calculated collected fraction of total emission signal, signal-to-noise ratios, and signal-to-bleed-through ratios of the FDMM filter sets. The numbers are generated from the spectra viewer program Semrock Searchlight. | | | | |
| --- | --- | --- | --- | --- |
| **Filter set** | **Collected fraction of total emission signal** | **Signal-to-noise ratio** | **Signal-to-bleedthrough ratio from indicated fluorochrome** | |
| DAPI | 29% | 6.5 | 1 | DAPI |
|  |  |  | 14 | Atto425 |
|  |  |  | > 1000 | AF488 |
|  |  |  | > 1000 | Cy3 |
|  |  |  | > 1000 | AF594 |
|  |  |  | > 1000 | PerCP-Cy5.5 |
| 425 | 16% | 7.9 | > 1000 | DAPI |
|  |  |  | 1 | Atto425 |
|  |  |  | > 1000 | AF488 |
|  |  |  | > 1000 | Cy3 |
|  |  |  | > 1000 | AF594 |
|  |  |  | > 1000 | PerCP-Cy5.5 |
| 488 | 34% | 6.4 | > 1000 | DAPI |
|  |  |  | 409 | Atto425 |
|  |  |  | 1 | AF488 |
|  |  |  | 383 | Cy3 |
|  |  |  | > 1000 | AF594 |
|  |  |  | > 1000 | PerCP-Cy5.5 |
| Cy3 | 40% | 6.4 | > 1000 | DAPI |
|  |  |  | > 1000 | Atto425 |
|  |  |  | 333 | AF488 |
|  |  |  | 1 | Cy3 |
|  |  |  | 40 | AF594 |
|  |  |  | > 1000 | PerCP-Cy5.5 |
| 594 | 33% | 2.3 | > 1000 | DAPI |
|  |  |  | > 1000 | Atto425 |
|  |  |  | > 1000 | AF488 |
|  |  |  | > 1000 | Cy3 |
|  |  |  | 1 | AF594 |
|  |  |  | > 1000 | PerCP-Cy5.5 |
| PerCP | 65% | 6.0 | > 1000 | DAPI |
|  |  |  | 342 | Atto425 |
|  |  |  | > 1000 | AF488 |
|  |  |  | > 1000 | Cy3 |
|  |  |  | 93 | AF594 |
|  |  |  | 1 | PerCP-Cy5.5 |
