# Supplementary material for: Filter-Dense Multicolor Microscopy
Source: PLoS One. 2015 Mar 4;10(3):e0119499. doi: 10.1371/journal.pone.0119499 (PMC4349739; doi:10.1371/journal.pone.0119499)
Supplement: S3 Table — (DOCX) [file pone.0119499.s009.docx]

| **Table S3.** Calculated collected fraction of total emission signal, signal-to-noise ratios, and signal-to-bleed-through ratios on standard filter sets from Chroma Technology Corp. The numbers are generated from the spectra viewer program Semrock Searchlight. | | | | |  |
| --- | --- | --- | --- | --- | --- |
| **Filter set** | **Collected fraction of total emission signal** | **Signal-to-noise ratio** | **Signal-to-bleedthrough ratio from indicated fluorochrome** | |  |
| DAPI | 36% | 8.6 | 1 | DAPI |  |
|  |  |  | 2234 | AF488 |  |
|  |  |  | >10 000 | Cy3 | |
|  |  |  | >10 000 | AF594 |  |
| 488 | 61% | 2.4 | >10 000 | DAPI |  |
| (39002) |  |  | 1 | AF488 |  |
|  |  |  | 31 | Cy3 |  |
|  |  |  | >10 000 | AF594 |  |
| Cy3 | 60% | 3.1 | >10 000 | DAPI |  |
| (49004) |  |  | 349 | AF488 |  |
|  |  |  | 1 | Cy3 |  |
|  |  |  | 0.9 | AF594 |  |
| 594 | 83% | 3.4 | >10 000 | DAPI |  |
| (49008) |  |  | 1981 | AF488 |  |
|  |  |  | 5 | Cy3 |  |
|  |  |  | 1 | AF594 |  |
