# Supplementary material for: Filter-Dense Multicolor Microscopy
Source: PLoS One. 2015 Mar 4;10(3):e0119499. doi: 10.1371/journal.pone.0119499 (PMC4349739; doi:10.1371/journal.pone.0119499)
Supplement: S4 Table — (DOCX) [file pone.0119499.s010.docx]

| **Table S4.** Calculated collected fraction of total emission signal, signal-to-noise ratios, and signal-to-bleed-through ratios on standard filter sets from Carl Zeiss Microscopy. The numbers are generated from the spectra viewer program Semrock Searchlight. | | | | |
| --- | --- | --- | --- | --- |
| **Filter set** | **Collected fraction of total emission signal** | **Signal-to-noise ratio** | **Signal-to-bleedthrough ratio from indicated fluorochrome** | |
| DAPI | 29% | 4.2 | 1 | DAPI |
|  |  |  | >10 000 | AF488 |
|  |  |  | >10 000 | Cy3 |
|  |  |  | >10 000 | AF594 |
| 488 | 77% | 2.6 | 6784 | DAPI |
| (FS 38) |  |  | 1 | AF488 |
|  |  |  | 102 | Cy3 |
|  |  |  | >10 000 | AF594 |
| Cy3 | 76% | 3.3 | >10 000 | DAPI |
| (FS 46HE) |  |  | 360 | AF488 |
|  |  |  | 1 | Cy3 |
|  |  |  | 1.1 | AF594 |
| 594 | 84% | 3.3 | >10 000 | DAPI |
| (FS 45) |  |  | 1956 | AF488 |
|  |  |  | 5 | Cy3 |
|  |  |  | 1 | AF594 |
